# Bilateral Anterior Uveitis Revealing Relapsing Polychondritis

**DOI:** 10.4274/tjo.galenos.2018.28909

**Published:** 2019-04-30

**Authors:** Murat Hasanreisoğlu, Hüseyin Baran Özdemir, Fulya Yaylacıoğlu, Mestan Ertop, Zeynep Aktaş

**Affiliations:** 1 Gazi University Faculty of Medicine, Department of Ophthalmology, Ankara, Turkey; 2 University of Health Sciences, Ulucanlar Eye Training and Research Hospital, Ophthalmology Clinic, Ankara, Turkey

**Keywords:** Anterior uveitis, relapsing polychondritis, systemic autoimmune disease

## Abstract

Relapsing polychondritis is a potentially lethal but rare systemic autoimmune disease. The major site of inflammation is the connective tissue, usually involving the ears, nose, larynx, tracheobronchial tree, and cardiovascular system. Although scleritis and episcleritis are known to be the most probable ocular manifestation, it may also present with uveitis. We present the case of a 22-year-old young lady who initially referred with bilateral red and painful eyes caused by anterior uveitis. Her right ear was also red and painful, consistent with cartilaginous inflammation. She was diagnosed with relapsing polychondritis with bilateral anterior uveitis and chondritis of the ear in conjunction with the rheumatology department. Bilateral anterior uveitis should evaluated and monitored carefully in patients with relapsing polychondritis.

## Introduction

Relapsing polychondritis (RP) is an idiopathic inflammatory disease of cartilaginous structures that can involve the ears, nose, larynx, tracheobronchial tree, and cardiovascular system.^1,2^ The latter are responsible for the high morbidity and mortality of the disease. The diagnostic criteria for RP are based on characteristic clinical manifestations.^1^

Ocular manifestations occur in approximately 60% of patients with RP.^3^ The most common manifestations are scleritis, episcleritis, keratitis, and conjunctivitis. Relapses and exacerbations are common. Uveitis occurs in approximately 25% of patients with RP, which is most commonly either in the form of anterior uveitis or a sclerouveitis.^4^ Proptosis, corneal perforation, retinal vasculitis, and optic neuritis leading to blindness are other possible ocular manifestations of RP.^4^ In this paper, we report a case with anterior uveitis as an ophthalmic manifestation of RP.

## Case Report

**Figure 1:**
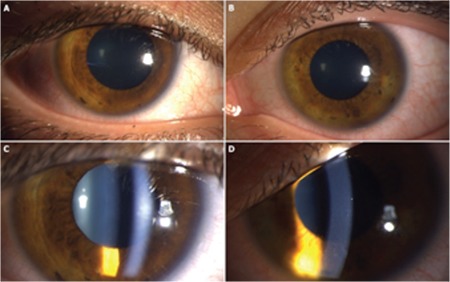
Anterior segment photography of the right eye (A) and left eye (B) at initial presentation showing bilateral conjunctival hyperemia. Slit-lamp photography of the right eye (C) and left eye (D) showing bilateral anterior chamber reaction and non-granulomatous keratic precipitates which were more prominent in the left eye

**Figure 2:**
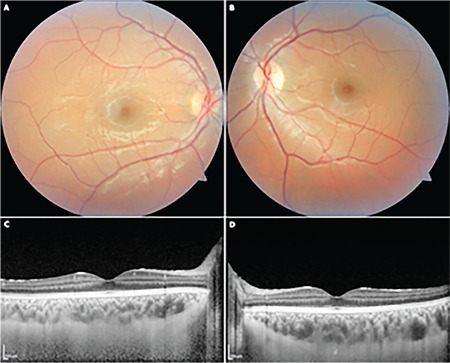
Fundus photography of the right eye (A) and left eye (B) with normal findings. Enhanced depth imaging spectral-domain optical coherence tomography of the right eye (C) and left eye (D) showing fovea and choroidal thickness, which was unaffected

**Figure 3:**
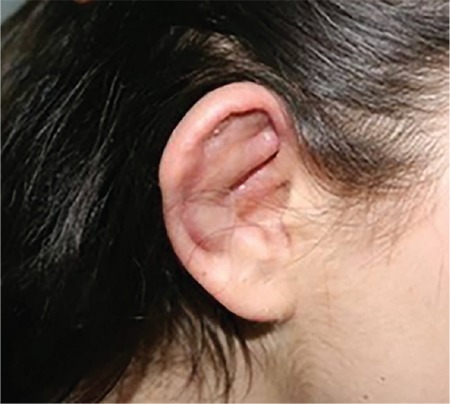
Right auricular chondritis as a presenting sign

A 22-year-old woman was referred to Gazi University, Department of Ophthalmology with photophobia and redness in both eyes starting one week earlier. Best corrected visual acuity was 20/20 in both eyes, although she described discomfort with her vision. Slit-lamp examination revealed bilateral conjunctival injection and anterior chamber reaction which was graded as +4 accompanied by fine, non-granulomatous bilateral keratic precipitates (Figure 1). Dilated fundus examination demonstrated normal retinal findings, with no vascular sheathing or any sign of retinitis (Figure 2). Optical coherence tomography (OCT), enhanced depth imaging-OCT, and fundus autofluorescence (FAF) were all normal (Figure 3). In addition to her ophthalmic symptoms, the patient had redness and pain in her right ear. Physical examination of the patient showed cartilaginous inflammation of the right ear (Figure 3). The patient was referred to the rheumatology department for further systemic evaluation. Hematological examination demonstrated elevated serum erythrocyte sedimentation rate and C-reactive protein level (69 mm/hr ve 126 mg/L, respectively). Complete blood count and other biochemical parameters were within normal ranges. Infective and inflammatory markers were also normal (anti-DNA, ANA, C3 and C4 immunoglobulin, anti-SSA, anti-SSB, anti-SM, anti-SCL, and anti-JO). The patient was treated with topical dexamethasone 0.1 mg/5 mL ophthalmic solution hourly, cyclopentolate 1 %3 times a day, and systemic oral 1 mg/kg/day prednisolone therapy with a plan to taper.

**Figure 4:**
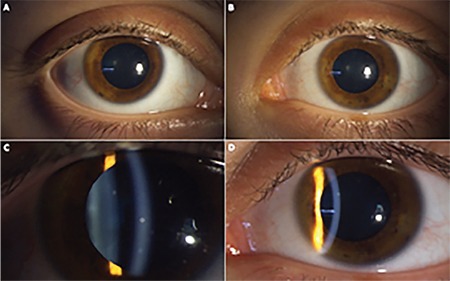
Anterior segment photography (A, C: right eye; B, D: left eye) after one month of treatment showing decrease of anterior chamber reaction with few keratic precipitates, trace anterior chamber reaction, and no synechia

After one month of this combination of topical and oral steroid therapy, her best corrected visual acuity was stable and visual deterioration was resolved. Slit-lamp biomicroscopy revealed a dramatic regression in the anterior chamber reaction, with only trace anterior chamber cells/flare and few keratic precipitates (Figure 4). Treatment continued with slow tapering.

## Discussion

RP is a rare autoimmune disorder characterized by recurrent episodes of inflammation involving cartilaginous structures containing type 2 collagen throughout the body, resulting in tissue damage and destruction.^1^ The disease causes repetitive inflammation mainly affecting the ears, nose, and tracheobronchial tract. Proteoglycan-rich structures throughout the body such as the joints, eyes, inner ear, blood vessels, heart, and kidneys may also be involved.^5^ The most common early signs of RP are auricular chondritis and polyarthritis, occurring in over 80% of patients.^6^ However, common symptoms are often absent in the early stages; therefore, it may mimic any other rheumatologic disease involving joint, ocular, cutaneous, or audio-vestibular dysfunction, resulting in diagnostic delays.

Ocular manifestations are found in 50-70% of patients and are of great importance since they are correlated with disease activity.^7^ Although two major ocular manifestations documented in the literature include episcleritis or scleritis, uveitis has also been reported in 3-22% of cases and can compromise visual outcomes.^7^ Uveitis, though uncommon, is reported to be anteriorly located and non-granulomatous. This is consistent with the present case, which presented with bilateral anterior uveitis concurrent with chondritis of one ear, the knees, and fingers. The diagnostic criteria for RP are based on characteristic clinical manifestations. Biopsy confirmation may be needed if the diagnosis is suspected. In our case, biopsy did not seem crucial, since the auricular chondritis was typical as an early manifestation of her disease. Although RP is most common in patients between the ages of 40 and 60,^1^ it can also affect young adults or children, as in the present case. The prognosis of patients with RP is also variable and depends on the organ involvement and response to treatment.^8^ Treatment is customized according to the severity and site of the disease. Mild forms are treated with anti-inflammatory and anti-neutrophilic agents but advanced cases, including those involving acute airway obstruction, multiple relapses, and cardiovascular disease, may require high doses of prednisone (1 mg/kg per day) or even intravenous pulse methylprednisolone.^6^ In our case, topical steroids and low-dose oral prednisolone treatment was administered initially. Because response to treatment was slow and the chondritis and uveitis were controlled only to a limited degree, the oral prednisolone dose was increased gradually to the maximum level of 1 mg/kg. This effectively reduced disease activity.

To sum up, RP is an inflammatory disease of unknown etiology that affects the connective tissue. The diagnosis is difficult to confirm and it is even more challenging to predict and manage disease progression. Ocular manifestations, although not rare, should be evaluated carefully, as relatively less common presentations such as uveitis can lead to vision loss if not detected and treated.
